# Clozapine Induces Agranulocytosis via Inflammatory and Hematopoietic Cytokine Induction of the JAK–STAT Signaling Pathway: Evidence From Network Pharmacology and Molecular Docking

**DOI:** 10.1111/cns.70206

**Published:** 2025-01-08

**Authors:** Ying Zhang, Ranli Li, Ximing Chen, Yachen Li, Qiuyu Zhang, Lei Yang, Lina Wang, Yun Sun, Fuqiang Mao, Chuan Jun Zhuo

**Affiliations:** ^1^ Computational Biology Center, Tianjin Anding Hospital Nankai University Affiliated Tianjin Anding Hospital, Tianjin Mental Health Center of Tianjin Medical University Tianjin China; ^2^ Department of Psychiatry and Psychology, School of Basic Medical Sciences Tianjin Medical University Tianjin China; ^3^ Laboratory of Psychiatric‐Neuroimaging‐Genetic and Co‐Morbidity (PGNP_Lab) Nankai University Affiliated Tianjin Anding Hospital, Tianjin Mental Health Center of Tianjin Medical University Tianjin China

**Keywords:** agranulocytosis, clozapine, molecular docking, network pharmacology

## Abstract

**Background:**

Clozapine exhibits significant therapeutic efficacy in schizophrenia, especially treatment‐resistant schizophrenia. However, clozapine can cause agranulocytosis, a fatal adverse effect, and the aim of this study is to explore this mechanism based on network pharmacology and molecular docking.

**Method:**

Six and two databases were used to identify targets associated with clozapine and agranulocytosis, respectively. The bioinformatics online platform was used to identify overlaps between the drug and disease targets. The protein–protein interaction (PPI) network was characterized using Cystoscope 3.10.1 and STRING. Kyoto Encyclopedia of Genes and Genomes (KEGG) and Gene Ontology (GO) were analyzed using the DAVID online platform. A drug‐target‐pathway‐disease network was constructed utilizing Cystoscope 3.10.1. The Auto Dock Vina and PyMOL software were used to verify the molecular docking of clozapine and core targets.

**Results:**

The analysis revealed 188 overlapping targets. The PPI and KEGG enrichment pathway analyses demonstrated that clozapine induces agranulocytosis by modulating the hematopoietic cell lineage and JAK–STAT signaling pathways via interleukin‐3 (IL3), IL6, IL2 receptor subunit alpha (IL2RA), and granulocyte colony‐stimulating factor. Binding energies between clozapine and core targets were favorable (< −7.0 kcal/mol).

**Conclusion:**

Clozapine‐induced agranulocytosis may be linked to the JAK–STAT inflammatory signaling pathway through inflammatory and hematopoietic‐related cytokines. Our findings enhance our comprehension of the potential mechanisms underlying clozapine‐induced agranulocytosis.

## Graphical abstract

To our knowledge, this is the first systematic investigation of the possible mechanisms of CIA using network pharmacology combined with molecular docking. Our findings suggest that clozapine induces agranulocytosis via the hematopoietic cell lineage, the JAK–STAT signaling pathway, and several targets, including IL3, IL6, IL2RA, and CSF3.

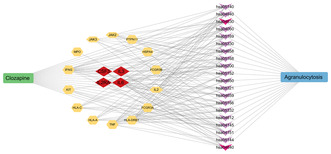

## 1 Introduction

Schizophrenia is a chronic, disabling disease [1, 2]. Evidence‐based first‐line treatments for schizophrenia include antipsychotic drugs [3]. Clozapine, which was discovered more than 65 years ago, is a second‐generation antipsychotic drug [2, 4]. Clozapine improves symptoms and reduces mortality and suicide [5, 6, 7]. Currently, clozapine is the only drug approved for treatment‐resistant schizophrenia [4]. However, most doctors are intimidated by clozapine because the mechanism of action is poorly understood [4] and clozapine incurs serious adverse reactions [3].

Agranulocytosis is caused by multiple drugs, including clozapine [3, 8, 9, 10, 11]. The incidence of agranulocytosis due to clozapine is 0.8%–1.0% [12, 13]. However, recent studies demonstrated that the risk of clozapine‐induced agranulocytosis (CIA) decreases over time, and clozapine can be used for long periods [14]. The molecular mechanisms of CIA were examined to provide a theoretical basis for the clinical use of clozapine. CIA is associated with human leukocyte antigens, such as HLA‐DQB1 and HLA‐B [15, 16, 17, 18, 19, 20, 21]. In addition, immune dysregulation, inflammatory reactions, and oxidative stress may contribute to CIA, but these mechanisms have not been systematically investigated [8, 22, 23]. Agranulocytosis is an unpredictable and potentially fatal adverse reaction to clozapine [9]. Therefore, an in‐depth systematic investigation into the molecular mechanisms of CIA is desperately needed.

Network pharmacology employs a computational approach that combines chemical structure with genomic data to systematically link compounds to pharmacological information using biological function data analysis and network construction [24]. Potential binding proteins for specific compounds are identified based on network analysis of the interactions between multiple targets and pathways [25, 26]. This approach can systematically predict the molecular mechanisms of potential drug or disease targets. Molecular docking technology is an essential and widespread approach to clarifying biological and molecular mechanisms and is used to assist in drug discovery tasks, including the prediction of adverse effects [27]. The active site for the compound and the target protein can be identified based on the “key and lock” pairing principle [28]. Experimental validation studies should be performed based on the results of the molecular docking analysis [28].

**FIGURE 1:**
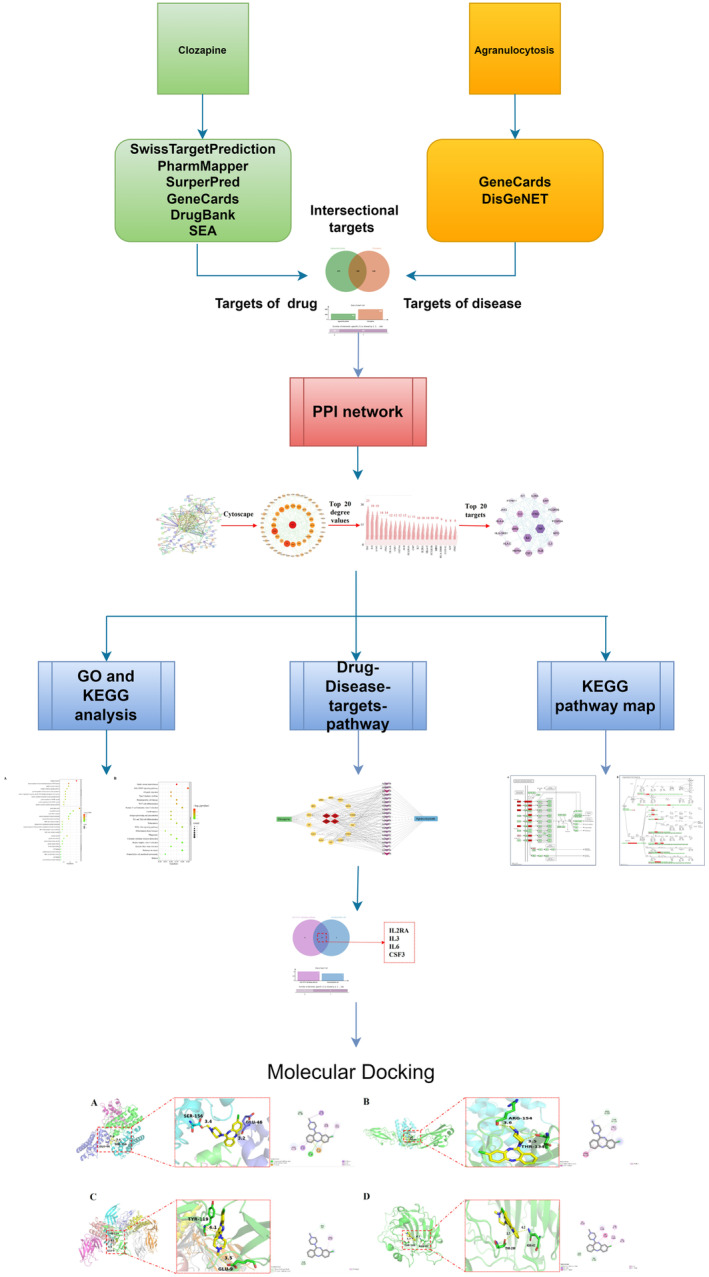
The flow diagram for the study. CSF, colony‐stimulating factor; IL3, interleukin‐3; IL6, interleukin‐6; IL2RA, interleukin‐2 receptor subunit alpha; GO, Gene Ontology; KEGG, Kyoto Encyclopedia of Genes and Genomes; PPI, protein–protein interaction.

Addressing the adverse effects of clozapine, including agranulocytosis, can enhance patient safety and reduce the risk of hospitalization due to infections and related complications. Furthermore, addressing the adverse effects of clozapine can improve treatment adherence and reduce anxiety among clinicians and patients [29], enhance the efficacy in patients with treatment‐resistant schizophrenia, and improve patient quality of life [30]. Addressing the adverse effects of clozapine can also reduce the considerable healthcare expenditures associated with infections, hospitalizations, antibiotic therapies, and routine hematological monitoring of agranulocytosis [31, 32]. In summary, addressing clozapine‐induced agranulocytosis will enhance patient safety, treatment adherence, and quality of life and facilitate the restoration of social functioning while alleviating the financial burden on both families and the healthcare system. However, the mechanism of CIA is complex and unclear at present. The objective of this study was to systematically determine the mechanisms of CIA using computational biology techniques. Figure 1 depicts the flowchart used in this present study, including searching targets, constructing networks, enrichment analysis, and molecular docking.

## 2 Methods

### 2.1 Obtaining Prospective Clozapine Targets

The chemical structure of clozapine was derived from PubChem [33] (https://pubchem.ncbi.nlm.nih.gov/). Generic names, SDF files, and canonical SMILES of clozapine were used to identify and predict the underlying targets by searching the Pharm Mapper [34] (http://www.lilab‐ecust.cn/pharmmapper/), the SwissTargetPrediction [35] (http://swisstargetprediction.ch/), SuperPred [36] (https://prediction.charite.de/), GeneCards [37] (https://www.genecards.org/), SEA [38] (https://sea.bkslab.org/), and DrugBank [39] (https://go.drugbank.com/) databases. Potential targets with *Z*‐scores ≥ 2 in the PharmMapper database and a probability > 0 in the SwissTarget Prediction database were screened. Known strong binders and potential targets were chosen from the SuperPred database. Protein‐coding genes from the GeneCards database were obtained. Using the SEA database, the standard target filter was set to a MaxTC > 1. The latent targets were converted into gene symbols for *Homo sapiens* utilizing the Uniprot [40] (https://www.uniprot.org/) database. Targets from the six databases were combined and duplicate targets were eliminated.

### 2.2 Acquisition of the Underlying Targets of Agranulocytosis

The keyword “agranulocytosis” was searched in the GeneCards and DisGeNET [41] (https://www.disgenet.org/home/) databases to obtain agranulocytosis‐related targets. GeneCards is an extensive database that covers genomics, transcriptomics, and proteomics [42]. DisGeNET encompasses both normal and aberrant genes in a range of human disorders [41]. targets were translated into gene symbols using the Uniprot database. Targets of the two databases were integrated, and duplicates were eliminated.

### 2.3 Identification of Common Drug‐Disease Targets

Targets associated with agranulocytosis and clozapine were researched using the Bioinformatics [43] (http://www.bioinformatics.com.cn/) online platform to obtain intersection targets. Overlaps between drug and disease targets were identified as possible targets for CIA.

### 2.4 Building a Protein–Protein Interaction Network and Analyzing the Major Targets

To investigate the common targets of clozapine and agranulocytosis, the overlapping targets were imported into the STRING [44] (https://cn.string‐db.org/) database. The targets were limited to “ *Homo sapiens* ” and filtered using a protein interaction score cut‐off of 0.7. Unconnected nodes were hidden in the network to get PPI information, which was input into Cytoscape 3.10.1 [45] (https://cytoscape.org/) to construct a PPI network. The topological parameters and degree values [46, 47, 48, 49] were used to deeply analyze the characteristics of the nodes in the interaction network. The node degree refers to the number of edges directly connected to a specific node [47]. In a protein–protein interaction (PPI) network, the node degree indicates the number of interactions a protein has with other proteins. A higher degree means that the protein interacts with many other proteins, suggesting that the protein plays a critical role in the network's functionality and biological processes (BP) [47, 48]. Conversely, a lower degree indicates fewer interactions, which suggests a more auxiliary role in the network.

### 2.5 Analysis of Key GO and KEGG Enrichment Targets

GO and KEGG enrichment targets were identified using the DAVID [50] (https://david.ncifcrf.gov/) online tool. GO enrichment categories include molecular function (MF), cellular components (CC), and biological processes. The first 10 items of MF, CC, and BP were selected. The top 20 KEGG enrichment analyses were selected. All gathered data were input into the bioinformatics and KEGG [51] (https://www.genome.jp/kegg/) online tools for visualization.

### 2.6 Drug‐Target‐Pathway‐Disease Network Construction

To determine the mechanism for CIA, a drug‐target‐pathway‐disease network was established using Cytoscape 3.10.1 software. The intersecting lines indicate relationships between biomolecules. The drug‐target‐pathway‐disease network facilitates an intuitive understanding of complicated relationships.

### 2.7 Molecular Docking

Molecular docking was used to verify clozapine‐target interactions and evaluate binding capacities. The first step was protein preparation. Candidate proteins with overlapping targets in significant signal pathways were regarded as core protein targets [52]. The crystal structures for the key targets were downloaded from the RCSB Protein Data Bank [53] (PDB, https://www.rcsb.org/). Initial ligands and water molecules were eliminated with Pymol (https://pymol.org/2/) software. The second step was ligand preparation. The 2D structure of clozapine was obtained from the PubChem database. The energy of the SDF file was minimized and transformed into a mol2 structure using Chem3D Ultra 14.0 (https://library.bath.ac.uk/chemistry‐software/chem3d) software. In the third step, the ligand and proteins were processed to be recognized by the AutoDock program. For instance, hydrogen bonds were added, charges were calculated, and atom types in receptor proteins were added. The root of the ligand and rotatable bonds were identified. Finally, the ligand and receptor were saved in PDBQT format using AutoDock 1.5.6. The fourth step was grid preparation. The receptor protein was central, the ligand was outside the docking box, and the docking box was entirely encased using AutoDock 1.5.6. The docking parameter files included the PDBQT format files with the receptor and ligand file names, the center coordinates of the docking grid, the grid size, and the output settings in the same folder. Molecular docking was implemented using AutoDock Vina [54]. According to the scoring function, lower scores indicated a greater binding affinity between ligands and receptors [55]. Pymol and Discovery Studio (https://discover.3ds.com/discovery‐studio‐visualizer) software were employed to visualize the 3D and 2D structures.

## 3 Result

### 3.1 Potential Targets of Clozapine and Agranulocytosis

**FIGURE 2:**
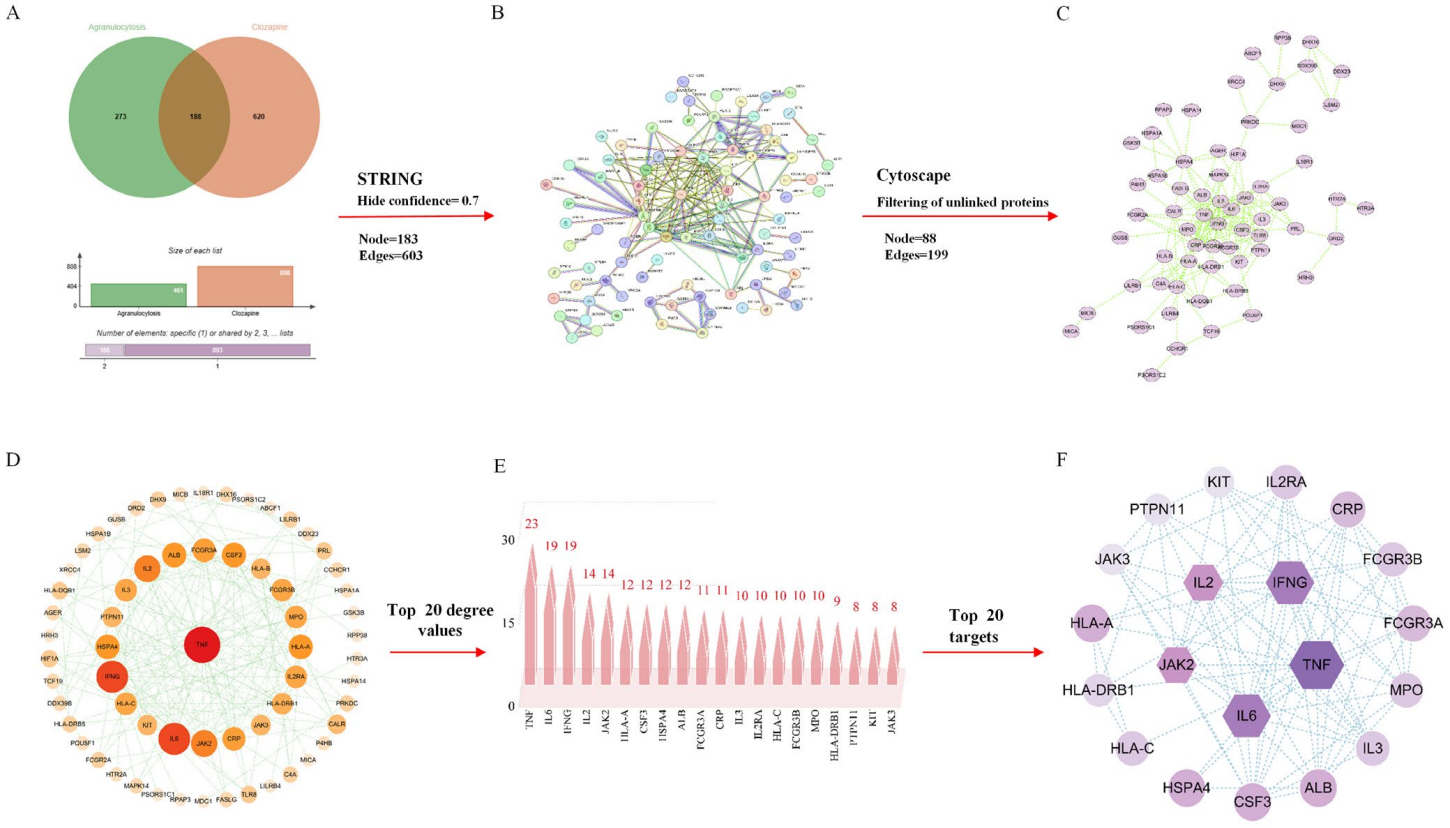
(A) The intersection of targets related to clozapine and agranulocytosis. (B and C) Construction of a PPI network of intersection targets. Nodes represent proteins. Edges represent protein–protein associations. (D and E) Topological screening procedure for the PPI network. The top 20 targets for clozapine‐induced agranulocytosis have the highest degree values. (F) Construction of a PPI network of core targets.

After combining and eliminating duplicates, 808 targets pertaining to clozapine were obtained from six databases. Similarly, 461 targets related to agranulocytosis were collected from the GeneCards and DisGeNET databases after combining and deleting repetitive targets. A Venn diagram was generated using the Bioinformatics online tool. Ultimately, 188 overlapping targets for the CIA were identified (Figure 2A).

### 3.2 PPI Network and Primary Targets

A PPI network of intersecting targets was generated using the STRING database. The information from the PPI network was imported into the Cytoscape 3.10.1 software for visualization. After eliminating proteins with no structures or no connections with other proteins, the PPI network included 63 nodes and 179 edges (Figure 2B–D). Larger nodes indicate a better probability of being a significant target (degree value is higher). The 20 targets with the greatest degree values were selected as the core targets of CIA (Figure 2E,F and Table 1).

**TABLE 1:** Detailed information about the 20 core targets.

Gene	Target name	Degree
TNF	Tumor necrosis factor	23
IL6	Interleukin 6	19
IFNG	Interferon gamma	19
IL2	Interleukin 2	14
JAK2	Janus kinase 2	14
HLA‐A	Major histocompatibility complex, Class I, A	12
CSF3	Colony‐stimulating factor 3	12
HSPA4	Heat shock protein family A (Hsp70) member 4	12
ALB	Albumin	12
FCGR3A	Fc gamma receptor IIIa	11
CRP	C‐reactive protein	11
IL3	Interleukin 3	10
IL2RA	Interleukin 2 receptor subunit alpha	10
HLA‐C	Major histocompatibility complex, Class I, C	10
FCGR3B	Fc fragment of IgG receptor IIIb	10
MPO	Myeloperoxidase	10
HLA‐DRB1	Major histocompatibility complex, Class II, DR beta 1	9
PTPN11	Protein tyrosine phosphatase non‐receptor Type 11	8
KIT	KIT proto‐oncogene, receptor tyrosine kinase	8
JAK3	Janus kinase 3	8

### 3.3 GO Enrichment Analyses

**FIGURE 3:**
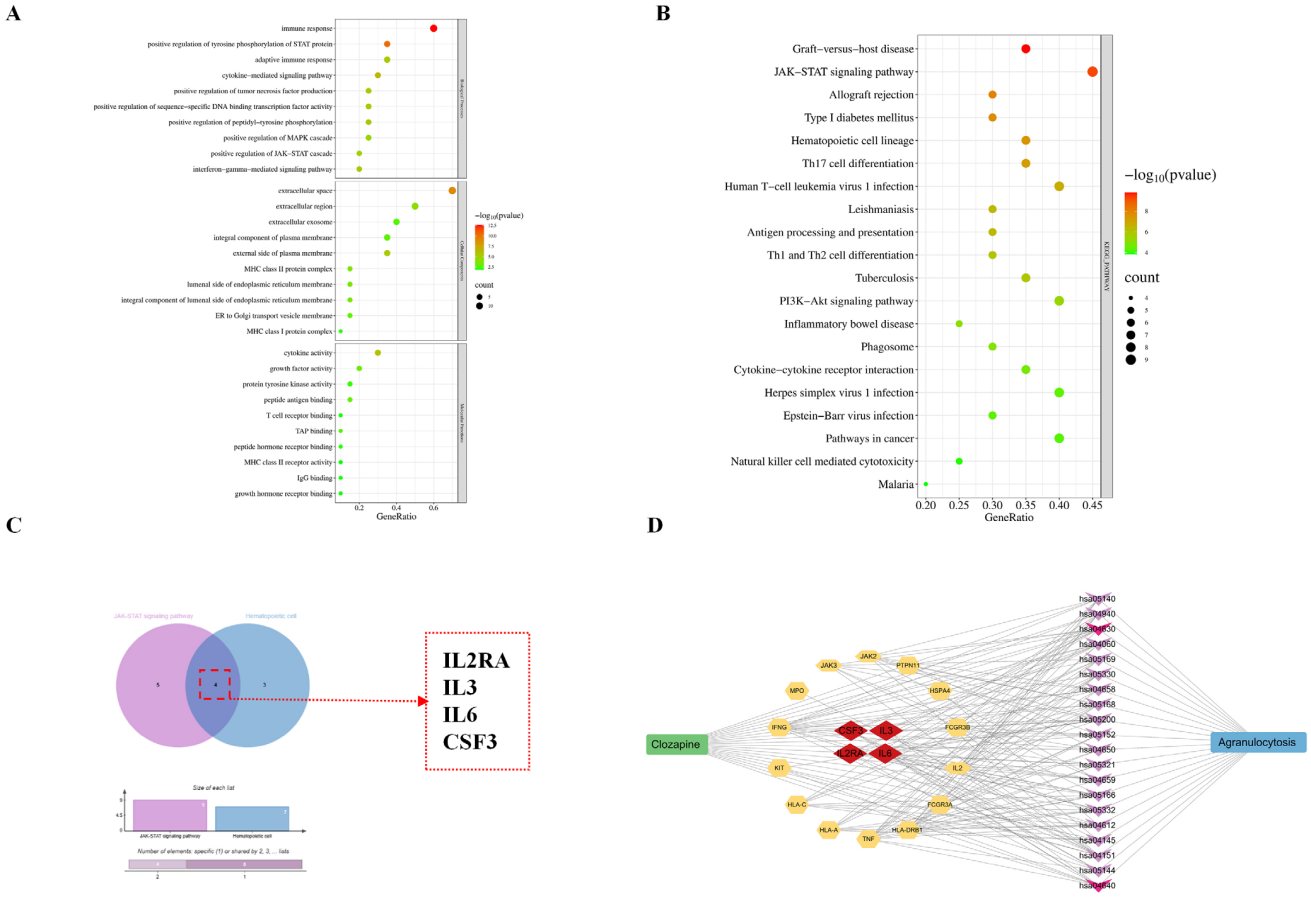
(A) GO enrichment analysis of the top 10 functions (*p* < 0.05). (B) KEGG pathway enrichment analysis of the top 20 pathways (*p* < 0.05). (C) The intersecting targets of the JAK–STAT signaling pathway and hematopoietic cell lineage are vital core targets. (D) Drug‐disease‐pathway target network. The green and blue round rectangles are expressed as drugs and diseases, respectively. The purple versus indicate pathways, and the pink versus indicate vital pathways. The yellow hexagons indicate targets, and the red diamonds indicate the crucial four core targets.

The top 20 core targets with high degree values were imported into the DAVID database for enrichment analysis. The 148 enrichment results included BP 116 items, 17 CC items, and 15 MF items. The top 10 entries of each GO enrichment type were chosen for visualization (Figure 3A). The identified BPs mainly involve immune response, positive regulation of tyrosine phosphorylation of STAT protein, and cytokine‐mediated signaling pathways. The CCs primarily involve extracellular space, extracellular region, and the external side of the plasma membrane. The MFs principally involve cytokine activity, growth factor activity, and protein tyrosine kinase activity.

### 3.4 KEGG Enrichment Pathway Analysis

**FIGURE 4:**
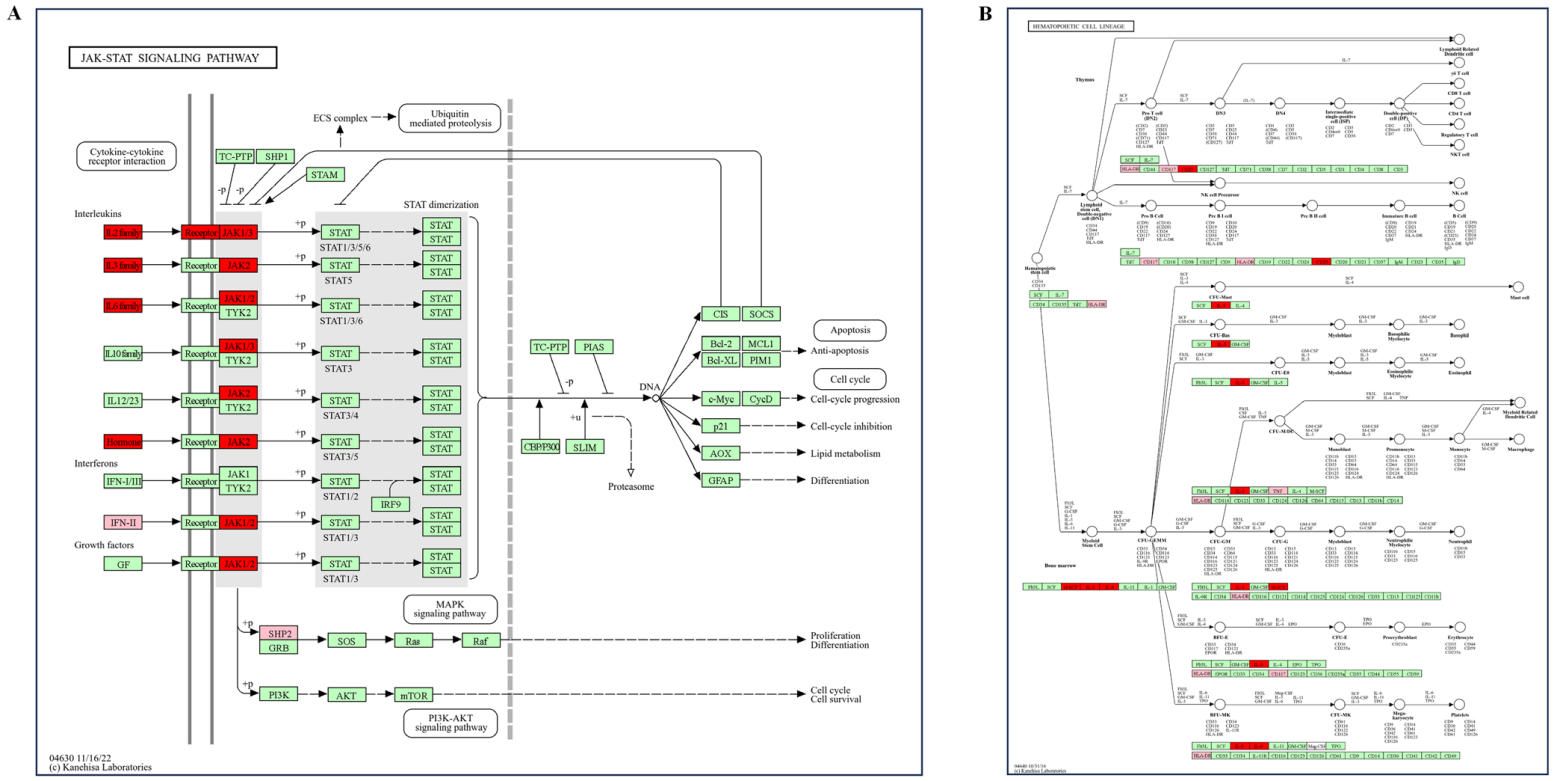
(A) JAK–STAT signaling pathway. (B) Hematopoietic cell lineage. Red rectangles represent potential targets of clozapine that lead to agranulocytosis.

The KEGG enrichment analysis of the 20 core overlapping targets resulted in 59 signaling pathways. Based on *p*‐values, the top 20 pathways were selected (Figure 3B and Table 2). The dominant signaling pathways were hematopoietic cell lineage and JAK–STAT signaling. The hematopoietic cell lines and JAK–STAT signaling pathway targets were imported into a bioinformatics online tool to identify common targets for molecular docking with clozapine (Figure 3C). The significant signaling pathways were selected for mapping (Figure 4A,B).

**TABLE 2:** Basic information about the KEGG enrichment results.

ID	Term	Count	*p*‐value
hsa05332	Graft‐versus‐host disease	7	1.61297E‐10
hsa04630	JAK–STAT signaling pathway	9	5.80836E‐10
hsa05330	Allograft rejection	6	1.02717E‐08
hsa04940	Type I diabetes mellitus	6	1.9575E‐08
hsa04640	Hematopoietic cell lineage	7	3.21811E‐08
hsa04659	Th17 cell differentiation	7	5.43659E‐08
hsa05166	Human T‐cell leukemia virus 1 infection	8	1.67772E‐07
hsa05140	Leishmaniasis	6	3.8495E‐07
hsa04612	Antigen processing and presentation	6	4.10798E‐07
hsa04658	Th1 and Th2 cell differentiation	6	9.40185E‐07
hsa05152	Tuberculosis	7	1.13129E‐06
hsa04151	PI3K‐Akt signaling pathway	8	4.30405E‐06
hsa05321	Inflammatory bowel disease	5	8.23346E‐06
hsa04145	Phagosome	6	1.12122E‐05
hsa04060	Cytokine‐cytokine receptor interaction	7	2.04812E‐05
hsa05168	Herpes simplex virus 1 infection	8	4.40484E‐05
hsa05169	Epstein–Barr virus infection	6	4.43494E‐05
hsa05200	Pathways in cancer	8	5.56842E‐05
hsa04650	Natural killer cell‐mediated cytotoxicity	5	0.000115932
hsa05144	Malaria	4	0.000139801

Abbreviation: KEGG, Kyoto Encyclopedia of Genes and Genomes.

### 3.5 Drug‐Target‐Pathway‐Disease Network

The key targets, clozapine, agranulocytosis, and the top 20 KEGG pathways were categorized and put into Cytoscape 3.10.1 to establish a drug‐target‐pathway‐disease network (Figure 3D). The green and blue round rectangles indicate clozapine and agranulocytosis, respectively. The purple versus indicate pathways, and the pink versus indicate vital pathways. The yellow hexagons indicate targets, and the red diamonds indicate the crucial four core targets. The results show that clozapine induces agranulocytosis through multiple targets and multiple signal pathways.

### 3.6 Molecular Docking of Clozapine With Core Targets

**FIGURE 5:**
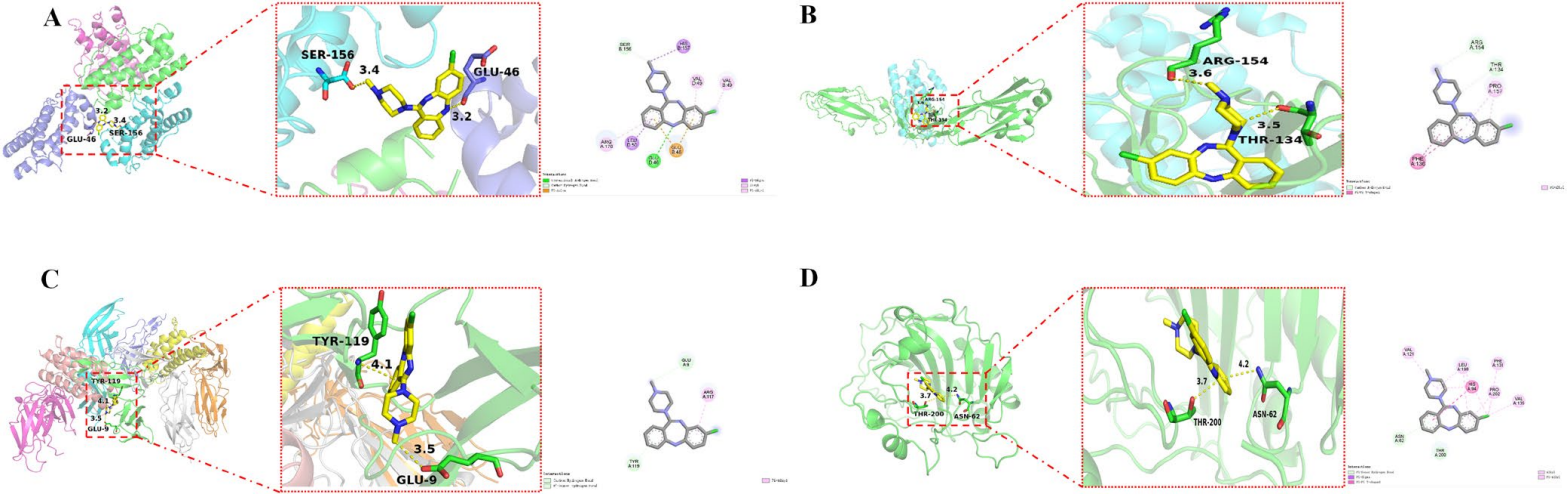
Molecular docking results for the main targets and clozapine, including (A) colony‐stimulating factor (CSF) 3 (5GW9), (B) interleukin (IL) 6 (1I1R), (C) IL2RA (2ERJ), and (D) IL3 (4PQ7).

The overlapping targets of the two significant signal pathways were identified as core targets, including IL3 (PDB ID: 4PQ7), IL6 (PDB ID: 1I1R), IL2RA (PDB ID: 2ERJ), and CSF3 (PDB ID: 5GW9). These core targets were subjected to molecular docking analysis with clozapine. A binding score < −7.0 kcal/mol is considered robust ligand‐receptor binding activity. The binding scores for clozapine docking with IL3, IL6, IL2RA, and CSF3 were good, especially CSF3. Clozapine created one hydrogen bond with SER156(B) and one hydrogen bond with GLU46(D) in CSF3, and the docking score was −9.7 kcal/mol. Clozapine created one hydrogen bond with ARG154(A) and one hydrogen bond with THR134(A) in IL6, and the docking score was −7.7 kcal/mol. Clozapine created one hydrogen bond with TYR119(A) and one hydrogen bond with GLU9(A) in IL2RA, and the docking score was −7.8 kcal/mol. Clozapine created one hydrogen bond with THR200(A) and one hydrogen bond with ASN62(A) in IL3, and the docking score was −8.4 kcal/mol. The 2D and 3D visualization of docking results and the detailed docking information are shown in Figure 5A–D and Table 3, respectively.

**TABLE 3:** Molecular docking of clozapine with the core target proteins.

Targets	PDB ID	Residue involved in H bonding	Binding energy (kcal/Mol)
CSF3	5GW9	GLU‐46, SER‐156	−9.7
IL2RA	2ERJ	GLU‐9, TYR‐119	−7.8
IL3	4PQ7	ASN‐62, THR‐ 200	−8.4
IL6	1I1R	ARG‐154, THR‐134	−7.7

Abbreviations: CSF3, Granulocyte colony‐stimulating factor; IL2RA, Interleukin‐2 receptor subunit alpha; IL3, Interleukin‐3; IL6, Interleukin‐6; PDB, Protein Data Bank.

## 4 Discussion

To our knowledge, this is the first systematic investigation of the possible mechanisms of CIA using network pharmacology combined with molecular docking. The bioinformatics analysis demonstrates that clozapine induces agranulocytosis via the hematopoietic cell lineage, the JAK–STAT signaling pathway, and several targets, including IL3, IL6, IL2RA, and CSF3. These results provide a theoretical foundation for in vitro experimental validation.

Using network analysis, we identified four core targets (IL2RA, IL6, CSF3, and IL3) that play a significant role in two crucial signal pathways. IL‐2 affects T cell development and differentiation and immune system regulation [56]. IL2RA/CD25, a subunit of IL2 receptor alpha, is essential for T cells [57]. T cells play an important role in autoimmune neutropenia in children [58]. Clozapine‐induced IL2RA effects in T‐cells may lead to agranulocytosis. IL6 is a cytokine primarily associated with inflammatory diseases and is involved in the immune response [59]. IL6 independently regulates granulopoiesis [60] and IL‐6 receptor blockers induce neutropenia [61]. Colony‐stimulating factor 3, also known as G‐CSF, is a glycoprotein [62] that stimulates the survival, proliferation, and differentiation of neutrophilic granulocytes and myeloid progenitor cells [63]. CSF3 levels may predict potential CIA [64] and CSF3 has potential therapeutic effects on severe CIA [65, 66, 67]. IL3 stimulates myeloid cell differentiation and proliferation, and IL3 receptors may play a role in granulocyte monocyte progenitor cell expression [68]. IL3 increases the absolute neutrophil count [69] and is associated with severe congenital neutropenia (SCN) [70]. Overall, the evidence supports the involvement of these multiple targets in CIA.

The GO enrichment analysis showed that immune response, positive regulation of tyrosine phosphorylation of STAT protein, extracellular space, cytokine activity, and protein tyrosine kinase activity were associated with CIA, in agreement with previous studies [64, 71, 72, 73, 74, 75]. Further investigation is required to validate these findings. The KEGG pathway analysis revealed that CIA is related to the hemopoietic system and inflammatory‐related signal pathways, including the hematopoietic cell lineage and the JAK–STAT signaling pathway. Maturation arrest of neutrophil precursors occurs early in myeloid differentiation [70]. Furthermore, patients with SCN exhibit impaired reactivity of primitive myeloid progenitor cells to early‐ or intermediate‐acting hematopoietic stimuli, including steel factor (SF), flk2/flt3 (FL), IL3, and G‐CSF [70]. Ogese et al. suggested that clozapine may cause neutrophil apoptosis by inducing specific T‐cell clones and altering the cytokine microenvironment [72]. In this context, we hypothesize that clozapine may cause abnormalities in primitive myeloid progenitor cells by affecting early‐ or mid‐acting hematopoietic factors and inflammatory signaling pathways, leading to agranulocytosis.

The four core targets identified in this study play important roles in controlling blood formation and immune responses [70, 76]. Moreover, clozapine changes a range of cell lineages, including myeloid cells, leading to abnormal blood markers [75]. The above evidence of molecular mechanisms and clinical indicators supports the findings of this study that hematopoietic cell lines are involved in CIA.

Mounting evidence supports the role of the inflammatory response in CIA [71, 73, 77]. Clozapine may act on the JAK–STAT signaling pathway by affecting the expression of protein molecules associated with signal transduction and T cells [72], which may induce CIA. Various cytokines, including the cytokines identified in this study, modulate the JAK–STAT signaling pathway, resulting in biological effects [72, 78, 79]. For example, G‐CSF mediates the JAK/STAT pathway through the negative regulatory factor SOCS3 [78]. IL3 receptors activate receptor‐associated JAKs, which affect the downstream βc signaling pathway [68, 79]. IL2 participates in the JAK3‐STAT5A signaling pathway to affect T cell proliferation [80]. Although our data is supported by the aforementioned studies, verification is required.

The molecular docking results confirmed the good affinities of clozapine for IL3, IL6, IL2RA, and CSF3. The range of docking scores for drugs and core targets was −7.7 to −9.7 kcal/mol. Thus, clozapine may regulate these targets and produce biological effects, leading to agranulocytosis. This study provides a fresh viewpoint on the molecular mechanism underlying CIA. However, this study has several drawbacks. First, some related targets have not been completely reported in the pharmacology database. Updating and comprehensively collecting targets in a timely manner will improve the accuracy of the results. Second, molecular interactions are intricate and multifaceted; thus, network pharmacology, which typically employs static and simplified models, may not accurately reflect the biological characteristics and mechanisms underlying these interactions. Consequently, additional research is needed to validate the findings of this study, particularly the kinetics, binding affinity, and mechanisms of interaction between proteins and pharmaceuticals. Third, even if our results clarify the role of hematopoietic‐related cytokines and JAK–STAT inflammatory signaling pathways in CIA, the mechanism for CIA is still not fully understood. Further studies should focus on how the JAK–STAT inflammatory signaling pathway is affected by clozapine, leading to agranulocytosis. Fourth, evidence generated from this approach is inferior to experimental validation. Consequently, pharmacological experiments are needed to verify these findings. Considerable progress is needed to translate the current research into clinical applications. For instance, can CIA be treated with anti‐inflammatory drugs? In summary, we employed in silico analysis methods for the first time to investigate the potential mechanisms underlying CIA, thereby providing fundamental insights into strategies to mitigate adverse drug reactions.

## 5 Conclusions

Our network pharmacology results suggest that clozapine regulates the hematopoietic cell lineage and JAK–STAT inflammatory signaling pathways via the following core targets: IL3, IL6, IL2RA, and CSF3. According to the molecular docking results, IL3, IL6, IL2RA, and CSF3 had good docking scores with clozapine. Our findings provide direction for further in‐depth studies of CIA. Given the limitations of in silico analysis and bioinformatics, additional pharmacological trials are needed to elucidate the mechanisms of CIA.

## Conflicts of Interest

The authors declare no conflicts of interest.

## Data Availability

Data sharing is not applicable to this article as no new data were created or analyzed in this study.
